# Strategic Variations in Fitts’ Task: Comparison of Healthy Older Adults and Cognitively Impaired Patients

**DOI:** 10.3389/fnagi.2016.00334

**Published:** 2017-01-20

**Authors:** Céline Poletti, Rita Sleimen-Malkoun, Leslie Marion Decker, Frédérique Retornaz, Patrick Lemaire, Jean-Jacques Temprado

**Affiliations:** ^1^Laboratoire de Psychologie Cognitive (LPC), Aix-Marseille Université, CNRSMarseille, France; ^2^Institut des Sciences du Mouvement (ISM), Aix-Marseille Université, CNRSMarseille, France; ^3^Unité COMETE, Université de Caen Normandie, INSERMCaen, France; ^4^Centre Gérontologique DépartementalMarseille, France

**Keywords:** aging, Fitts’ task, sensorimotor strategies, cognitive declines, information processing speed, executive functions

## Abstract

The present study aimed at investigating how healthy older adults (HOA) and cognitively impaired patients (CIP) differ in a discrete Fitts’ aiming task. Four levels of task difficulty were used, resulting from the simultaneous manipulation of the size of the target and its distance from home position. We found that movement times (MTs) followed Fitts’ law in both HOA and CIP, with the latter being significantly slower and more affected by increased task difficulty. Moreover, correlation analyses suggest that lower information processing speed (IPS) and deficits in executive functions (EFs) are associated with decline of sensorimotor performance in Fitts’ task. Analyses of strategic variations showed that HOA and CIP differed in strategy repertoire (which strategies they used), strategy distribution (i.e., how often they used each available strategy), and strategy execution (i.e., how quick they were with each available strategy). These findings further our understanding of how strategic variations used in a sensorimotor task are affected by cognitive impairment in older adults.

## Introduction

Declines of cognitive (e.g., Salthouse, 1996; Bashore et al., 1997) and motor (e.g., Rey-Robert et al., 2012; Temprado et al., 2013) performance are widely used in the literature to assess the effects of aging in the neuro-behavioral system. However, qualitative changes (i.e., the strategies used to adapt to task constraints) may also provide functionally meaningful information about age-related alterations of brain and cognition. During the last decade, the effects of aging on strategic variations have been widely investigated in a variety of cognitive tasks (for an overview see Lemaire, 2010, 2015). In this literature, a strategy is defined as “a procedure or a set of procedures used to achieve a higher level goal” (Lemaire and Reder, 1999, p. 365). Accordingly, Lemaire and Siegler (1995) proposed to distinguish between four main strategy dimensions: namely strategy repertoire (i.e., which strategies are used?), strategy distribution (i.e., how often each available strategy is used?), strategy execution (i.e., how quickly and accurately each strategy is applied?), and strategy selection (i.e., how do people choose among available strategies on each item?).

Recently, we have undertaken to extend this framework to the sensorimotor domain using the Fitts’ rapid-aiming task (Poletti et al., 2015, 2016). We made this choice since control processes used during aiming movement in Fitts’ task are representative of those used in several motor tasks (e.g., Abbs et al., 1984; Duysens and Van de Crommert, 1998; Collins and De Luca, 1993; Van de Crommert et al., 1998). It consists of performing rapid-aiming movements from a starting position toward a target, leading participants to adopt an optimal compromise between speed and accuracy (Elliott et al., 2001; Ketcham et al., 2002). The width (W) and/or distance (D) of the target can be varied to modulate task difficulty (Fitts, 1954; Fitts and Peterson, 1964). In our previous studies, the different strategies used by participants were inferred from the analysis of kinematic profiles of the aiming movements in the different task conditions (i.e., different index of difficulties, IDs). Specifically, on the basis of the type of sub-movement observed in the kinematic profile of the hand trajectory (i.e., no sub-movements, Type 1, 2 and 3 sub-movements), four different strategies were distinguished (i.e., the one-shot, overshoot, undershoot and progressive-deceleration strategies, respectively) that participants used to reach the target (and to manage the speed-accuracy trade-off). When using the one-shot strategy, participants directly reached the target without making additional corrective sub-movements. When using the overshoot strategy, participants performed a movement that brought their arm after the target and then had to make a secondary corrective sub-movement by reversing direction to reach the target. When using the undershoot strategy, participants performed a movement that fall short before the target and had to make a secondary corrective sub-movement (a reacceleration) to reach the target. When using the progressive deceleration strategy, participants performed a movement that brought their arm near the target and then slowly approached the target, leading to a lengthening of the deceleration phase.

Based on this methodology, we showed that these kinematic patterns fulfill the currently accepted criteria to empirically distinguish among strategies, namely: (a) main effect of strategies on collected measures (e.g., performance); (b) interaction effects between the strategy factor and one or several factors on strategy use and strategy performance; and (c) age-related differences in strategy use and performance (see Poletti et al., 2015). We empirically documented the usefulness of a strategy approach to further our understanding of processes underlying age-related changes in sensorimotor performance. For instance, we found that older adults used fewer strategies than young adults, thereby suggesting aging changes participants’ strategy repertoire. With respect to strategy distribution, we found that older adults predominantly used strategies that involve corrective sub-movements (i.e., the undershoot and progressive deceleration strategies), whereas young adults preferred the strategy without corrective sub-movements (i.e., the one-shot strategy). Finally, our results showed that older adults were slower than young adults whatever the strategies they used. Therefore, we concluded that, on the one hand, age-related differences in performance are associated with strategies that young and older participants used to perform the task (Poletti et al., 2015), and on the other hand, the alterations of executive functions (EFs), combined with greater processing demands to perform the task, resulted in age-related differences in strategy repertoire and strategy selection (Poletti et al., 2016).

Our previous studies were carried out with healthy young and older participants. However, it has been pointed out in the literature that performance declines and strategic variations were dramatically affected in persons with degenerative disorders, such as patients suffering from mild cognitive impairment or Alzheimer’s disease (AD). In the cognitive domain, several studies used the strategy perspective to compare performance’ declines during healthy and cognitively impaired older adults (e.g., Duverne et al., 2003; Gandini et al., 2009). For instance, during a simple subtraction problem solving task, Arnaud et al. (2008) have shown that healthy older adults (HOA) and AD patients used the same two strategies (i.e., retrieval and non-retrieval strategies) with equal proportions, but differed in strategy execution (i.e., AD patients were slower than HOA, especially on harder problems). However, in a numerosity estimation task, Gandini et al. (2009) have shown that HOA and AD patients differed both in the use and in the execution of the different numerosity estimation strategies. Indeed, AD patients used the easier visual estimation strategy more often than HOA (75% vs. 62%), and they were impaired in strategy execution when using the harder anchoring strategy.

Mild cognitive impairment and AD patients also currently exhibit significant deficits in their motor performance (Ott et al., 1995; Hirono et al., 1997). Motor alterations resulting from cognitive impairments have been predominantly investigated in locomotion (Montero-Odasso et al., 2012; Amboni et al., 2013; Montero-Odasso and Hachinski, 2014). In upper-limb movements, previous research has suggested that age-related changes in motor behavior (e.g., movement slowing, decreased kinematic smoothness; see Yan et al., 1998, 2000; Yan, 2000) are exacerbated in patients suffering from Mild cognitive impairment or AD (Carrasco et al., 2000; Yan et al., 2008). Yan et al. (2008) reported that participants with mild or severe cognitive impairments show a reduction in the smoothness, the coordination, and the consistency of handwriting movements, suggesting that compromised motor performance could be one of the behavioral manifestations of cognitive impairments (see Scarmeas et al., 2004; Eggermont et al., 2010; Maquet et al., 2010; Mirelman et al., 2015 for supporting this hypothesis). However, to our knowledge, the effects of cognitive impairments on strategic variations of behavior in aiming tasks have never been investigated. The present study addressed this issue by comparing strategic variations in healthy and cognitively impaired older adults, in a Fitts’ task. Since EFs and processing speed are seriously affected in cognitively impaired patients (CIP), one can expect at least an amplification of these variations generally observed in HOA. Indeed, it has been suggested that EFs are strongly involved in Fitts’ task, especially during the deceleration phase of the movement (see Rey-Robert et al., 2012; Sleimen-Malkoun et al., 2013; Temprado et al., 2013).

To test this general hypothesis, we first determined whether Fitts’ law was respected in both groups of participants, as it is a prerequisite to analyze strategic variations. Accordingly, we examined the efficiency function (i.e., movement times (MTs) as a function of the difficulty of the task) in HOA and in CIP. We expected to observe longer MTs and steeper slopes in CIP than in HOA. Moreover, we wanted to examine the role of EFs and information processing speed (IPS) on participants’ performance by correlating participants’ slopes with scores on specific neuropsychological tests. Second, we analyzed group differences in strategic variations. Concerning strategy repertoire, we predicted that CIP would use fewer strategies than HOA, starting from lower IDs. Additionally, we expected that HOA and CIP would exhibit different strategy preferences and strategy distributions. As a result of declines in EFs and IPS, we predicted that CIP would be more dependent on the visually guided deceleration phase, which would lead them to use most frequently strategies with sub-movement corrections (i.e., more than HOA). Also, for both groups, strategy selection should be influenced by task difficulty. Finally, we analyzed strategy performance as a function of task difficulty in each group. We predicted that group differences in MTs would depend on the strategy used and would be larger for strategies with sub-movement corrections.

## Materials and Methods

### Participants

Twenty-one right-handed participants (11 HOA; *mean age* = 73.6 years; *age range*: 66–89 and 10 CIP; *mean age* = 81.7 years; *age range*: 65–90), voluntarily participated to the study. HOA lived independently and were recruited from the community of Marseille, whereas CIP were on long-term stay nursing home residents at the Centre Gérontologique Départemental of Marseille. All participants had no history of neurological (e.g., Parkinson’s disease, stroke) or psychiatric disorders (e.g., schizophrenia, bipolar disorder) or alcohol and substance abuse. Moreover, they had no uncorrected visual impairment and did not suffer from any upper-limb dysfunctions or pain that could affect the achievement of the task. They were unfamiliar with the task and the apparatus.

We collected information about each participant’s socio-demographic characteristics (i.e., age, gender and years of formal education). Then, to ensure the construct group consistency all participants completed a battery of questionnaires assessing their functional status. The battery consisted of the Katz’s Index of Independence in Activities of Daily Living Scale-6 items (Katz, 1983), the Lawton’s Instrumental Activities of Daily Living Scale-4 items (Lawton and Brody, 1969), the life-space mobility (UAB Life-Space Assessment; Baker et al., 2003; Auger et al., 2009), the self-reported falls in the past year, and the degree of fear of falling (French version of the Falls Efficacy Scale-International; Tinetti et al., 1990). In addition, they all underwent neuropsychological testing to assess depression (Short Form of the Geriatric Depression Scale; Sheikh and Yesavage, 1986), general cognitive function (Montreal Cognitive Assessment; Nasreddine et al., 2005), processing speed (Digit Symbol-Coding, and Symbol Search, subtests of the Wechsler Adult Intelligence Scale-Third Edition; Wechsler, 1997), episodic memory (Free and Cued Selective Reminding Test; Buschke, 1984), working memory (Digit Span subtest of the Wechsler Adult Intelligence Scale-Third, Forward and Backward; Wechsler, 1997), verbal fluency (Category and Letter Fluency Tests; Robert et al., 1998), inhibition (Victoria Stroop Test; Bayard et al., 2011) and cognitive flexibility (Trail Making Test; Vazzana et al., 2010).

We ensured that none of the HOA obtained a score lower than 26 on Montreal Cognitive Assessment, lower than six on Index of Independence in Activities of Daily Living Scale, lower than four on Instrumental Activities of Daily Living Scale, and higher than six on Short Form of the Geriatric Depression Scale, in order to be included in the current study. Sociodemographic characteristics, self-reported functional status, and neuropsychological scores are shown in Table 1. As can be seen by the *p*-value of the *t*-test, the two groups are significantly different for the sociodemographic characteristics (except for gender, *p* > 0.05), the functional status (except for Index of Independence in Activities of Daily Living Scale, *p* > 0.05), and the neuropsychological status (except for the Short Form of the Geriatric Depression Scale, *p* > 0.05). After a presentation of the experiment, each participant signed an informed written consent, approved by the local ethic committee of Aix-Marseille University, in accordance with the ethical standards laid down in the Declaration of Helsinki.

**Table 1:** **Sociodemographic characteristics, self-reported functional status and neuropsychological scores for healthy older adults (HOA) and cognitively impaired patients (CIP)**.

	HOA	CIP	*p*-value
**Sociodemographic characteristics**
Age (years)	73.6 ± 6.4	81.7± 9.0	*p* < 0.05
Number of men (women)	5/16	2 (8)	ns
Years of education	13.0 ± 2.7	7.6± 3.2	*p* < 0.001
**Functional status**
Index of independence in activities of daily living^1^	6.0 ± 0	5.6± 1.0	ns
Instrumental activities of daily living scale^2^	4.0 ± 0	3.1± 0.9	*p* < 0.01
Life-space mobility	96.2 ± 17.0	46.3± 17.3	*p* < 0.001
Self-reported falls in the past year	0.4 ± 0.7	2.0± 2.0	*p* < 0.05
Fear of falling	19.6 ± 1.8	30.4± 13.7	*p* < 0.01
**Neuropsychological status**
Depression
Short form of the geriatric depression scale^3^	2.7 ± 1.4	4.6± 4.2	ns
General cognitive function
Montreal cognitive assessment^4^	28.6 ± 1.2	24.2± 3.5	*p* < 0.001
Information processing speed
Digit symbol-coding^5^	64.6 ± 11.2	37.2± 8.6	*p* < 0.001
Symbol search^6^	28.1 ± 5.0	16.9± 3.8	*p* < 0.001
Episodic memory
Free and cued selective reminding test—Immediate free recall^7^	35.8 ± 5.9	25.4± 7.1	*p* < 0.01
Free and cued selective reminding test—Delayed free recall^7^	13.8 ± 1.5	10.5± 3.6	*p* < 0.05
Working memory
Digit span-forward^8^	11.3 ± 1.6	8.5± 1.6	*p* < 0.001
Digit span-backward^8^	8.3 ± 2.0	6.0± 1.1	*p* < 0.01
Verbal fluency
Category fluency^9^	24.4 ± 5.2	15.7± 4.6	*p* < 0.001
Letter fluency^9^	17.6 ± 2.7	11.5± 2.6	*p* < 0.001
Inhibition
Victoria stroop test (I-C/C)^10^	1.2 ± 0.4	2.3± 1.5	*p* < 0.05
Cognitive flexibility
Trail making test^11^	16.9 ± 10.1	74.7± 41.6	*p* < 0.001

*Note. ^1^Katz’s Index of Independence in Activities of Daily Living Scale, ^2^Lawton’s Instrumental Activities of Daily Living Scale, ^3^0–4: normal, 5–9: mild depression, 10–15: more severe depression, ^4^A score of 26 or above is considered normal, ^5^Number of items correctly completed within the time limit of 120 s, ^6^Number of correct minus incorrect responses within the time limit of 120 s, ^7^Number of items recalled, ^8^Number of items (digit lists) correctly repeated, ^9^Number of words correctly produced, ^10^Time needed to perform Victoria Stroop Test Interference minus the time needed for Victoria Stroop Test Color divided by the time needed for Victoria Stroop Test Color, ^11^Time needed to perform Trail Making Test (part B) minus the time needed for Trail Making Test (part A)*.

### Apparatus and Procedure

Participants were comfortably seated on a chair at a height-adjusted table, with their dominant hand resting on their lap and their non-dominant forearm resting on a USB digitizer (Wacom Intuos4 XL; 1024 × 768 pixel resolution) positioned on the tabletop right in front of them, in portrait orientation. The digitizer was connected to a portable PC (Dell, Latitude-D420). Using a hand-held non-marking stylus (Wacom, Generation 2 tip sensor), participants were asked to perform discrete rapid-aiming movements, from an initial home position towards a target. The home position, represented as a red circle of 0.5 cm diameter, was always aligned with the center of the target, which was represented by a red horizontal rectangle (17.4 cm × various width values). Home and target positions were printed on a white paper sheet and inserted under the transparent plastic film cover of the digitizer. Aiming movements involved shoulder flexion and elbow extension with full stops on home position (movement initiation) and on target position (movement termination). To avoid trunk compensations, participants were required to keep their belly pressed against the table.

In this experiment, the task’s ID was scaled via the manipulation of both the size of the target and its distance from home position. Four ID levels, ranging from 3 to 6 bits by increments of 1 bit were used. The different ID conditions are detailed in Table 2. The order of presentation of the conditions was randomized in-between participants.

**Table 2:** **Distance and width parameters characterizing index of difficulty (ID) conditions**.

ID condition (bits)	Distance (cm)	Width (cm)
3	8	2.0
4	12	1.6
5	16	1.2
6	20	0.8

Before each trial, participants were asked to hold still the stylus on the home position until they heard a beep. They were free to initiate their movements at any time following the onset of the beep and were informed that it was not necessary to minimize reaction times. However, they were firmly instructed to keep optimal speed-accuracy trade-off that is “to move as fast as possible from the home position to the target position and to stop on it”. Before each ID condition, participants were allowed to complete three (unrecorded) familiarization trials. Then, they were requested to perform four blocks of 16 trials each, for a total of 64 trials. To prevent fatigue, a short rest was allowed between each trial and each block. For each ID condition, the allowed error rate was 12.5% (maximum 2 trials out of 16). If more than two trials were missed, the missed trials were repeated at the end of the condition.

### Variables and Data Processing

The pen-tip raw displacement data were recorded using a customized software (ICE) developed at the laboratory (Institute of Movement Sciences, Marseille, France) at a sampling frequency of 250 Hz. The recorded data were filtered using a second-order dual pass (no phase-lag) Butterworth filter with a cutoff frequency of 10 Hz. First, second and third derivatives of displacement (velocity, acceleration and jerk, respectively) were then computed in MATLAB (MathWorks, v.7.5.0 R2007b). Movement onset and offset were determined on the basis of velocity profiles using the optimal algorithm of Teasdale et al. (1993). The critical velocity threshold was obtained by multiplying peak velocity by 0.05.

First, this procedure allowed us to calculate for each trial, in each condition MTs that correspond to the times to reach the target. MTs were then used to calculate *efficiency functions*. Namely, we analyzed the ID-MT relationships using simple linear regression models carried out on mean group values. Second, to check whether the prescribed IDs were respected or not (Sleimen-Malkoun et al., 2012), the effective target width (We) was calculated from the standard deviation of movement end points (MacKenzie, 1992) using the following formula: We = 2*1.96*SD_A_, where SD_A_ is the standard deviation of movement amplitude (i.e., the effective distance), and 1.96 is the boundary of a normal distribution at 95%. Then, we compared the distributions of movement end points (centered on mean movement amplitude and bounded by calculated We) and the prescribed ones (centered on target distance and bounded by target edges). These comparisons yielded no significant statistical differences (*t*s < 1). As a consequence, the prescribed ID levels were used for all participants.

Kinematic analyses were also carried out. Specifically, primary and secondary sub-movements were identified by following a procedure currently used in the literature and previously used by Poletti et al. (2015) in a comparable study. The primary sub-movement was characterized by a rapid movement that brings the limb near the target and the optional secondary sub-movement corresponded to an on-line control phase during which the target is approached (Woodworth, 1899). Then, following Meyer et al. (1988), the end of the *primary sub-movement* was defined as the moment of time when one of the following three events occurred after the velocity reaches its peak: (a) the velocity crossed zero, changing from positive to negative; (b) the acceleration crossed zero, changing from negative to positive; or (c) the jerk crossed zero, changing from positive to negative. This procedure enabled us to calculate kinematic variables that were related to the type of secondary sub-movement identified (see Dounskaia et al., 2005). Each secondary sub-movement has been used to distinguish types of strategies.

Three of the four available strategies that participants used to reach the target have been detected in this experiment. The undershoot strategy included re-acceleration towards the target; the progressive-deceleration strategy referred to an increase of the deceleration phase, and the one-shot strategy consisted in reaching the target with no corrections. The overshoot strategy, previously identified in Poletti et al. (2015) study, and corresponding to corrections for overshooting was never used by any of the participants in this study.

## Results

All trials containing errors (9.6%) were removed (analyses percentages of errors revealed only a main effect of ID, *F*_(3,60)_ = 14.11, *MS*e = 68.34, $\eta_{\text{p}}^{2}$ = 0.41). Moreover, one patient was excluded from the analyses since her performance did not follow Fitts’ law. Unless otherwise noted, all reported effects are significant with *p* < 0.05.

### Group Differences in Movement Times

To check if Fitts’ law was respected, MTs were first analyzed^1^ with a two-factor Group (HOA, CIP) × ID (3, 4, 5, 6 bits) ANOVA, with repeated measures on the last factor. Then, to determine the efficiency function, ID-MT relation was analyzed using a simple linear regression model carried out on mean group values. The slopes of the different efficiency functions estimated information-processing capacities in each age group (in bits/s). Student’s *t*-statistic was used to test for significant group differences in slopes.

MTs were significantly larger in CIP than in HOA (663 ms vs. 529 ms; *F*_(1,18)_ = 16.55, *MS*e = 21, 308, $\eta_{\text{p}}^{2}$ = 0.48) and increased with ID scaling (506 ms, 557 ms, 616 ms, and 704 ms; *F*_(3,54)_ = 116.90, *MS*e = 1215, $\eta_{\text{p}}^{2}$ = 0.87). The Group × ID interaction was significant, *F*_(3,54)_ = 4.83, *MS*e = 1215, $\eta_{\text{p}}^{2}$ = 0.17, resulting from a larger increase in MTs as a function of increased IDs in CIP (556 ms, 621 ms, 683 ms and 790 ms, respectively) than in HOA (457 ms, 493 ms, 549 ms and 617 ms, respectively).

**Figure 1:**
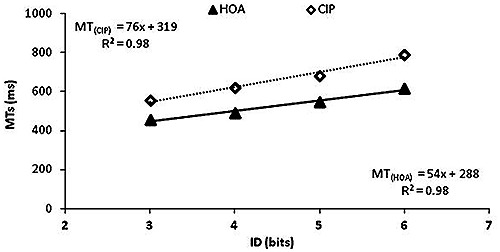
**Efficiency functions in both groups.** Efficiency functions of cognitively impaired patients (CIP; open diamonds) showed a larger slope than that of healthy older adults (HOA; black triangles).

Efficiency functions resulting from linear fittings of the ID-MT relation in each group, along with corresponding equations, are displayed in Figure 1. Fitts’ law was found to account for at least 96% of changes in MTs. CIP slope’s was larger than that of HOA (76 and 54, respectively; *t* = 2.53, *p* < 0.05). This result showed a multiplicative slowing in CIP, with a slowing rate of 41%.

### Relationships between the Slopes of Fitts’ Law and Scores on Neuropsychological Tests

We conducted Pearson partial correlation, controlling for age group and years of formal education, to examine the role of EFs and IPS on participants’ performance. Table 3 summarizes partial correlations between mean participants’ slopes and scores on the following neuropsychological tests: (a) Trail Making Test; (b) Victoria Stroop Test; (c) Digit Symbol Coding Test; and (d) Symbol Search Test. Participants’ slopes were positively correlated with the Victoria Stroop Test, and negatively correlated with the Digit Symbol Coding Test and Symbol Search Test. In this table, we included also one composite measure (i.e., Index) of EFs and one composite measure IPS. Composite measures were calculated by averaging the *z* scores of Trail Making Test and Victoria Stroop Test performance on the one hand, and of Digit Symbol Coding Test and Symbol Search Test performance on the other hand, for EFs and IPS, respectively. Higher index of EFs and lower index of IPS corresponded to a decreased performance whereas lower index of EFs and higher index of IPS corresponded to an increased performance. Participants’ slopes were positively correlated with index of EFs, as increased scores in EF tests were linked to a poorer performance. Moreover, they were negatively correlated with index of IPS, as decreased scores in IPS tests were linked to a poorer performance.

**Table 3:** **Correlation matrix for the full sample (*n* = 20) in participants’ slopes**.

Variables	1	2	3	4	5	6	7
1– Slope	1.00	0.45	0.50*	−0.61**	−0.54*
	−0.67**	0.62**
2– Trail making test		1.00	0.17	−0.29	−0.42	−0.42	0.70***
3– Victoria stroop test			1.00	−0.29	−0.44	−0.43	0.83***
4– Digit symbol coding test				1.00	0.46	0.84***	−0.38
5– Symbol search test					1.00	0.87***	−0.56*
6– Index of IPS						1.00	−0.56*
7– Index of EFs							1.00

Note. *p < 0.05; **p < 0.01; ***p < 0.001. Index was calculated as follows: Index of IPS = mean of scores *z* of Digit Symbol Coding Test and Symbol Search Test performance, Index of EFs = mean of scores z of Trail Making Test and Victoria Stroop Test performance.

In sum, the present results showed that MTs followed Fitts’ law in both HOA and CIP. Moreover, correlation analyses suggest that slower IPS and altered EFs are associated with poorer sensorimotor performance in the Fitts’ task.

### The Role of Executive Functions and Information Processing Speed in Group Differences in Slopes

To assess mediational effects of executive control and IPS between groups and slopes, we compared the proportion of variance accounted for by group (as reflected in increments in *R*^2^ corresponding to squared semi-partial correlations) before and after the variance associated with a composite measure of EFs and IPS were controlled. For example, the influence of variations in EFs on effects of group on slopes can be determined by comparing the proportion of variance associated with group before and after index of EFs was controlled.

Examination of the values (see Table 4) revealed that *R*^2^ associated with group for slopes was 0.35, and was reduced to 0.08 after controlling EFs. Thus, the group-related variance was reduced by 77% (i.e., [*R*^2^ with age alone − *R*^2^ partial]/*R*^2^ with age alone]; *Sobel test* = 2.41, *p* < 0.05). Group effect was no longer significant after controlling for index of EFs, *t*_(17)_ = 1.46, *p* = 0.16. Additional analyses showed that *R*^2^ associated with group was reduced to 0.18 after controlling index of IPS. Thus, the group-related variance was reduced by 49% (*Sobel test* = 2.01, *p* < 0.05), but group effect was still significant after controlling for index of IPS, *t*_(17)_ = 2.19, *p* = 0.04.

**Table 4:** **Percent attenuation of group variance in slope after control of executive functions and processing speed measures**.

	*R*^2^ (%)	Percent attenuation
Group	35	-
Group IPS	18	49
Group EFs	8	77

In sum, group differences in slopes can be accounted for entirely by group-related decrease in efficiency of EFs, as EFs mediated the relation between groups and slopes.

### Group Differences in Strategy Repertoire

Examination of kinematic profiles revealed that both HOA and CIP used three strategies (i.e., one-shot, undershoot and progressive-deceleration strategies). The overshoot strategy, which is generally rarely used (see Poletti et al., 2015, 2016), was never used by any participant of the two groups. In the subsequent analyses, in order to simplify the presentation of data and to increase the number of observations in each condition, we combined the easier and harder IDs respectively, hence yielding two different levels of difficulty: “easy” (3 and 4 bits) and “hard” (5 and 6 bits). Also, one HOA participant was excluded for presenting outliers values exceeding the mean by more than two standard deviations.

**Figure 2:**
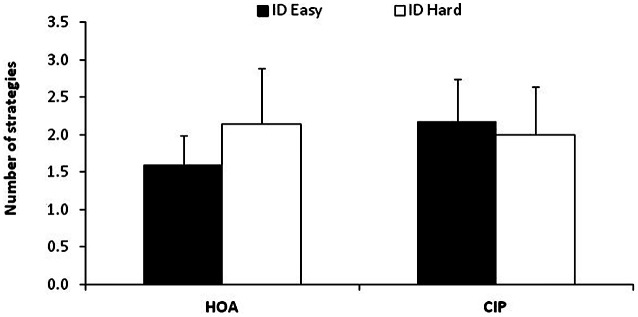
**Numbers of used strategies in both difficulty conditions for both groups.** Error bars represent Standard Deviations. The number of strategies increased with difficulty HOA but not in CIP.

To examine group differences in strategy repertoire^2^, mean number of strategies used by individuals were analyzed with a two-factor ANOVA: Group (2: HOA, CIP) × Difficulty (2: easy, hard), with repeated measures on the last factor. The Group × Difficulty interaction was significant (*F*_(1,36)_ = 10.04, *MS*e = 0.24, $\eta_{\text{p}}^{2}$ = 0.22). Planned comparisons showed that, HOA used a smaller number of strategies than CIP for easier IDs (1.6 vs. 2.2, *F*_(1,36)_ = 7.16; see Figure 2), whereas both groups used the same number of strategies for harder IDs (2.2 vs. 2.0, *F* < 1). This resulted from the fact that HOA increased the number of strategies used as a function of difficulty (*F*_(1,36)_ = 12.48), but not CIP (*F* = 11.03, *p* > 0.05).

### Group Differences in Strategy Distribution

To analyze group differences in strategy distribution, a three-way ANOVA (Group × Difficulty × Strategy), with repeated measures on Difficulty (2: easy, hard) and Strategy (2: one-shot, progressive-deceleration), was carried out on mean percentages of use of the two most often used strategies. The undershoot strategy, which was used on only 10% of trials, was excluded from this analysis to break the dependence between cells. On average, participants used the one-shot strategy (58%) more often than the progressive-deceleration strategy (32%).

**Figure 3:**
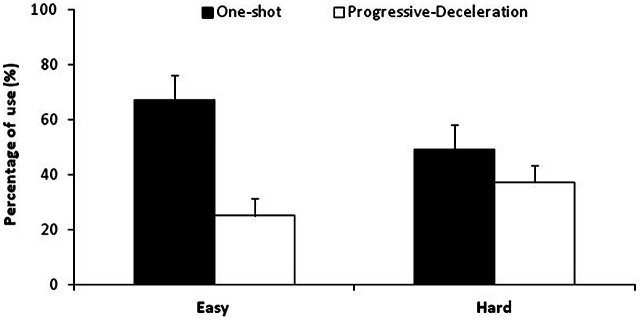
**Mean percentages of the one-shot and progressive deceleration strategies as function of task difficulty.** Error bars represent Standard Deviations. Percentages of use of the one-shot strategy were larger than that of progressive-deceleration strategy on easier trials but similar on harder trials.

As shown in Figure 3, analyses also revealed a significant Strategy × Difficulty interaction (*F*_(1,17)_ = 26.95, *MS*e = 159.8, $\eta_{\text{p}}^{2}$ = 0.61). Planned comparison showed that participants used more frequently the one-shot strategy than the progressive-deceleration strategy (67% vs. 25%, *F*_(1,17)_ = 23.86,) on easier trials, and these two strategies equally often on harder trials (49% vs. 37%, *F* = 1.24, *p* > 0.05).

### Group Differences in Strategy Execution

**Figure 4:**
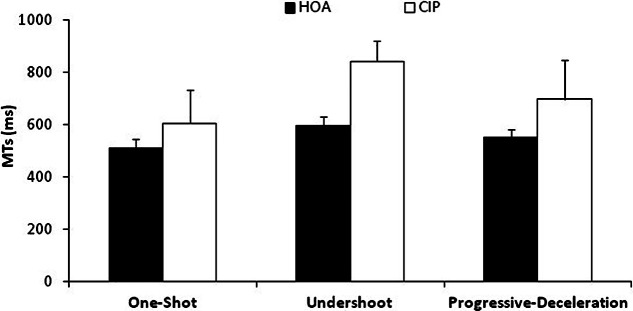
**Mean movement times (MTs) in both groups while using the one-shot, undershoot or progressive-deceleration strategies.** Error bars represent Standard Deviations. HOA were faster than CIP whatever strategy they used.

To analyze group differences in strategy execution, a two-way ANOVA (Group × Strategy), with repeated measures on the last two factors was carried out on MTs. As can be seen in Figure 4, analyses of MTs revealed that HOA were faster than CIP (553 ms vs. 706 ms; *F*_(1,17)_ = 20.46, *MS*e = 16, 225, $\eta_{\text{p}}^{2}$ = 0.55). A main effect of strategy was found (*F*_(2,34)_ = 62.06, *MS*e = 2160, $\eta_{\text{p}}^{2}$ = 0.79). Pairwise comparisons revealed that participants were faster with the one-shot strategy than with the progressive-deceleration strategy (*F*_(1,17)_ = 58.73), or than with the undershoot strategy (*F*_(1,17)_ = 84.77). Also, participants were faster with the progressive-deceleration strategy than with the undershoot strategy (*F*_(1,17)_ = 36.74). The Group × Strategy interaction was also significant (*F*_(2,34)_ = 15.70, *MS*e = 2160, $\eta_{\text{p}}^{2}$ = 0.48). Planned comparisons showed that HOA were the fastest with the one-shot strategy (511 ms). They were equally fast when using the undershoot and the progressive-deceleration strategies (596 ms and 552 ms, respectively). CIP were the fastest with the one-shot strategy (593 ms), and they were the slowest with the undershoot strategy (842 ms), the progressive-deceleration strategy being in the middle (682 ms). Although HOA were faster than CIP whatever strategy they used (*F*s > 9.82), differences between the two groups were the largest with the undershoot strategy (246 ms) and with the progressive-deceleration strategy (130 ms); they were the smallest with the one-shot strategy (82 ms).

## Discussion

The present study aimed at determining whether group differences in strategic variations might explain, at least partly, differences in sensorimotor performance between individuals with cognitive impairments and HOA. In the following, we discuss our findings with regards to observed group differences and their possible underlying processes.

### Group Differences in Performance

As a prerequisite, we first assessed quantitative changes associated with normal and pathological aging effects on MTs and efficiency functions in Fitts’ aiming task. Preliminary analyses showed group differences in all neuropsychological tests that were administered (except for the test assessing depression). Given that IPS and EFs are both involved in this Fitts’ task, and that HOA and CIP differed on tests assessing these functions, it can be hypothesized that they could have contributed to the emergence of group differences.

The CIP showed longer MTs than the HOA. These results are consistent with those reported by Goldman et al. (1999) and Yan et al. (2008). Analyses of the slopes of efficiency functions revealed steeper slope in CIP than in HOA, thereby suggesting decreased information processing rates of about 41% in CIP relative to HOA. According to our previous studies comparing young and older adults in the theoretical context of information theory (e.g., Temprado et al., 2013; Poletti et al., 2015), these results suggest that deficits in EFs mediate the observed effects of cognitive impairment on quantitative performance in Fitts’ task. Correlation analyses showing that participants’ slopes were positively correlated with the composite measure of EFs is consistent with this hypothesis. Most interesting and original in this study, analyses of strategic variations provide important and novel insights on the underlying mechanisms responsible for changes in motor behavior in CIP.

### Groups Differences in Strategic Variations

Our results showed that the number of strategies differed between groups of participants and depended upon trial difficulty. Specifically, HOA used fewer strategies on lower IDs relative to higher IDs. In contrast, CIP used an equal number of strategies for both lower and higher IDs. That is, HOA adults were able to focus on a smaller strategy repertoire (and inhibit most cognitively demanding strategies, those requiring corrective sub-movements) on lower IDs and increase the number of strategies on higher IDs. In contrast, CIP used all types of strategies on both lower and higher IDs. This suggests that they may have been unable to focus on using the strategies with no-corrective sub-movements (e.g., one-shot strategy) and to inhibit most demanding available strategies including corrective sub-movements (e.g., progressive-deceleration). Consistent with this, CIP used the progressive-deceleration strategy more often than HOA on lower IDs. One possibility is that given their decreased EFs, CIP were unable to inhibit strategies with corrective sub-movements like the progressive-deceleration on lower IDs in order to focus on strategies like one-shot that are easy to execute on lower IDs (and efficient). This interpretation is suggested by participants’ performance on neuro-psychological tests that showed deficits in EFs of CIP (see Diamond, 2013 for converging evidence). ANOVA on mean number of strategies using an index of EFs as covariates revealed that the effect of group was no longer significant after statistical control of EFs.

As in our previous studies (see Poletti et al., 2015, 2016), in the present experiment, participants used three different strategies (i.e., the one-shot, undershoot and progressive-deceleration strategies). We expected to observe: (a) more frequent use of strategies involving sub-movements, in particular for higher IDs; and (b) a difference between HOA and CIP in the use of corrective sub-movements’ strategy due to declines of EFs and IPS in the latter group. Surprisingly however, all participants favored the strategy without sub-movement (i.e., the one-shot strategy) over the strategy involving sub-movements (i.e., the progressive-deceleration strategies). This result could be seen as inconsistent with observations reported in other studies that is, more dependence on the cognitively guided deceleration phase, which leads elderly people to favor strategies including corrective sub-movements (Pratt et al., 1994; Poletti et al., 2015). This discrepancy might result from the chosen way to scale task difficulty in the different studies. Indeed, in the present experiment, ID was increased by manipulating both the size and the distance of targets, yielding task conditions with lower accuracy constraints than those used in other studies where only one of the target’s properties was manipulated at a time (see Poletti et al., 2015). However, despite the increased use of the one-shot strategy, our results were consistent with our predictions concerning the effect of ID and the effect of cognitive impairments on sub-movement strategy use. Indeed, with increasing task difficulty, participants tended to use more often the strategy involving corrective sub-movements. This choice reflected the emphasis on response accuracy and, likely more dependence on executive/cognitive control of movement execution when the task becomes difficult. Conversely, when the task was easier, participants were more able to calibrate the initial impulse of their movement and then placed more emphasis on response speed. It is noteworthy that the dominance of the one-shot strategy was observed for the HOA, while participants with cognitive impairments tended to use the one-shot equally often as the progressive-deceleration strategy. One can speculate that this observation is suggestive of more deficient impulse control processes in CIP. As expected, the two-way ANOVA (Group × Difficulty) showed that CIP were shorter in the percentage covered in the primary sub-movement relative to MTs for both ID conditions compared to HOA (*F* > 4.58).

Another set of interesting findings observed in the present study concerns strategy execution. To analyze execution, we classified the strategies as function of their presumed difficulty, with the most difficult strategy to execute being the one requiring longer MTs. Accordingly, our data revealed that the different strategies used by HOA and CIP to accomplish this Fitts’ task differed in relative difficulty. First, we found that the hierarchy of relative strategy difficulty was different in HOA and CIP. Indeed, HOA were the fastest with the one-shot strategy, and they were equally fast when using the undershoot and progressive-deceleration strategies. Moreover, CIP were the fastest with the one-shot strategy and the slowest with the undershoot strategy, the progressive-deceleration strategy being in-between. In addition, we observed that group differences were larger for undershoot strategy than for the one-shot strategy. This result is consistent with previous research investigating strategy execution in young and older adults (Poletti et al., 2015, 2016). Indeed, presumably, when participants used the undershoot strategy more executive processes were involved in the execution, leading CIP to be more impaired in their motor performance.

### Conclusion and Perspectives

Overall, the present study documented changes in strategic behaviors underlying differences in HOA and CIP performance in Fitts’ task. Our findings demonstrated the existence of significant groups differences in strategy repertoire, strategy distribution, and strategy execution. These findings suggest a critical role of EFs in this type of task. Interestingly, the strategic variations observed in the present study seem to follow general and common principles as in the cognitive domain. The origins of these commonalities deserve further investigations.

## Author Contributions

CP analyzed the data and contributed to the write-up of the manuscript. LMD collected the data and contributed to defining the experimental design. FR helped in screening participants and contributed to the manuscript write-up. RS-M, J-JT and PL contributed to data interpretation and manuscript write-up. All the authors approved the final manuscript.

## Funding

This research was supported by grants from the Agence Nationale de la Recherche (Grant# ANR-13-BSH2-0005-01 and Grant# ANR-2010-BLAN-1912-01).

## Conflict of Interest Statement

The authors declare that the research was conducted in the absence of any commercial or financial relationships that could be construed as a potential conflict of interest.

## Footnotes

^1^Analyses examining group differences in MTs were also performed with age and years of formal education factors as a covariate, and the same results came out significant.

^2^All the analyses examining group differences in strategies were also performed with age and years of formal education factors as a covariate, and the same results came out significant.

## Abbreviations

AD: Alzheimer’s disease
CIP: Cognitively impaired patient
EF: Executive function
HOA: Healthy older adult
ID: Index of difficulty
IPS: Information processing speed
MT: Movement time.
